# *catena*-Poly[[triphenyl­tin(IV)]-μ-2-(cyclo­hexyl­amino­carbon­yl)benzoato-κ^2^ *O* ^1^:*O* ^2^]

**DOI:** 10.1107/S1600536810026978

**Published:** 2010-07-14

**Authors:** Imtiaz-ud-Din, Raqiqt-ul-Rasool, Moazzam H. Bhatti, Seik Weng Ng

**Affiliations:** a Department of Chemistry, Allama Iqbal Open University, H/8 Islamabad, Pakistan; b Department of Chemistry, University of Malaya, 50603 Kuala Lumpur, Malaysia

## Abstract

In the title polymeric complex, [Sn(C_6_H_5_)_3_(C_14_H_16_NO_3_)]_*n*_, adjacent triphenyl­tin cations are bridged by the *N*-cyclo­hexyl­phthalamate anion through the carboxyl­ate and carbonyl O atoms, forming a helical chain running along the *b* axis. The amide N atom is a hydrogen-bond donor to the uncoordinated carboxyl­ate O atom. The geometry at the five-coordinate Sn atom is *trans*-C_3_SnO_2_ trigonal-bipyramidal.

## Related literature

For a review on organotin carboxyl­ates, see: Tiekink (1991 ▶, 1994 ▶). Triphenyl­tin aryl­carboxyl­ates generally exist as monomeric mol­ecules; see: Ng *et al.* (1986 ▶). For the synthesis of *N*-cyclo­hexyl­phthalamic acid, see: Dolzhenko *et al.* (2003 ▶).
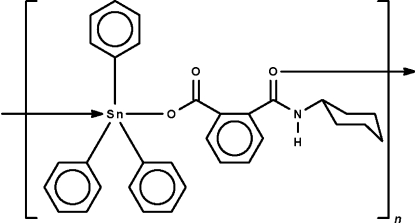


## Experimental

### 

#### Crystal data

[Sn(C_6_H_5_)_3_(C_14_H_16_NO_3_)]
*M* *_r_* = 596.27
Monoclinic, 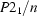

*a* = 9.8574 (5) Å
*b* = 16.0734 (8) Å
*c* = 17.1669 (8) Å
β = 99.447 (1)°
*V* = 2683.1 (2) Å^3^
*Z* = 4
Mo *K*α radiation
μ = 0.99 mm^−1^
*T* = 100 K
0.40 × 0.30 × 0.20 mm

#### Data collection

Bruker SMART APEX diffractometer
Absorption correction: multi-scan (*SADABS*; Sheldrick, 1996 ▶) *T*_min_ = 0.694, *T*_max_ = 0.827
25326 measured reflections
6165 independent reflections
5710 reflections with *I* > 2σ(*I*)
*R*_int_ = 0.023

#### Refinement

*R*[*F* ^2^ > 2σ(*F* ^2^)] = 0.019
*wR*(*F* ^2^) = 0.049
*S* = 1.02
6165 reflections
338 parameters
1 restraint
H atoms treated by a mixture of independent and constrained refinement
Δρ_max_ = 0.35 e Å^−3^
Δρ_min_ = −0.50 e Å^−3^

### 

Data collection: *APEX2* (Bruker, 2009 ▶); cell refinement: *SAINT* (Bruker, 2009 ▶); data reduction: *SAINT*; program(s) used to solve structure: *SHELXS97* (Sheldrick, 2008 ▶); program(s) used to refine structure: *SHELXL97* (Sheldrick, 2008 ▶); molecular graphics: *X-SEED* (Barbour, 2001 ▶); software used to prepare material for publication: *publCIF* (Westrip, 2010 ▶).

## Supplementary Material

Crystal structure: contains datablocks global, I. DOI: 10.1107/S1600536810026978/xu2796sup1.cif

Structure factors: contains datablocks I. DOI: 10.1107/S1600536810026978/xu2796Isup2.hkl

Additional supplementary materials:  crystallographic information; 3D view; checkCIF report

## Figures and Tables

**Table:** 

Sn1—C1	2.130 (1)
Sn1—C7	2.129 (2)
Sn1—C13	2.119 (1)
Sn1—O1	2.149 (1)
Sn1—O3^i^	2.392 (1)

**Table:** 

O1—Sn1—O3^i^	174.06 (4)

Symmetry code: (i) 
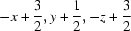
.

**Table 2:** Hydrogen-bond geometry (Å, °)

*D*—H⋯*A*	*D*—H	H⋯*A*	*D*⋯*A*	*D*—H⋯*A*
N1—H1⋯O2	0.86 (1)	1.85 (1)	2.666 (2)	158 (2)

**Fig. 1.**
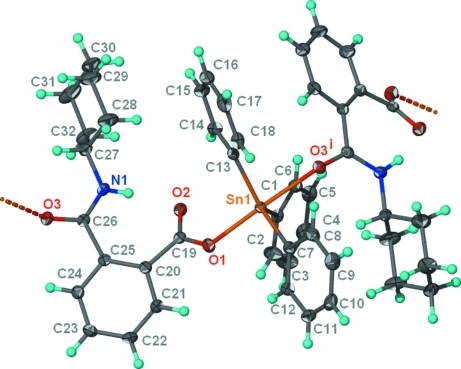
Thermal ellipsoid plot (Barbour, 2001) of a portion of polymeric Sn(C6H5)3(C14H16NO3) at the 70% probability level; hydrogen atoms are drawn as spheres of arbitrary radius.

## supplementary crystallographic information

### Comment

Triphenyltin aryl-carboxylates commonly exist as four-coordinate tetrahedral compounds unlike the alkyl-carboxylates, which adopt carboxylate-bridged polymeric chains structures (Ng *et al.*, 1986). Occasionally, the aryl-carboxylate anion bears a potential donor unit such as, in the present case, an amido group; such a donor group can interact with adjacent molecules to generate polymeric chain motifs (Tiekink, 1991; 1994). In the present *N*-cyclohexylphthalimic acid derivative (Scheme I), the amido oxygen atom engages in bonding to generate a chain motif (Fig. 1). The tin atom is displaced out of the C_3_Sn girdle in the direction of the covalently-bonded oxygen atom by 0.200 (1) Å; the Sn–O_covalent_ bond is significantly shorter than the Sn–O_dative_ bond.

### Experimental

*N*-Cyclohexylphthalamic acid was synthesized from the reaction of phthalic anhydride and cyclohexylamine in ethyl acetate by using a reported procedure (Dolzhenko *et al.*, 2003).

Triphenyltin hydroxide (1 mmol, 0.37 g) and *N*-cyclohexylphthalamic acid (1 mmol, 0.25 g) were heated in toluene (50 ml) for 6 h in a Dean-Stark water-separator. The solvent was then removed and the solid material recrystallized from a chloroform and *n*-hexane (3:1) mixture to furnish crystals.

### Refinement

Carbon-bound H-atoms were placed in calculated positions (C—H 0.95 to 0.98 Å) and were included in the refinement in the riding model approximation, with *U*(H) set to 1.2*U*(C).

The amido H-atom was located in a difference Fourier map, and was refined with N–H 0.86±0.01 Å; its temperature factor was freely refined.

### Crystal data

**Table:**

[Sn(C_6_H_5_)_3_(C_14_H_16_NO_3_)]	*F*(000) = 1216
*M**_r_* = 596.27	*D*_x_ = 1.476 Mg m^−^^3^
Monoclinic, *P*2_1_/*n*	Mo *K*α radiation, λ = 0.71073 Å
Hall symbol: -P 2yn	Cell parameters from 9925 reflections
*a* = 9.8574 (5) Å	θ = 2.4–28.3°
*b* = 16.0734 (8) Å	µ = 0.99 mm^−^^1^
*c* = 17.1669 (8) Å	*T* = 100 K
β = 99.447 (1)°	Block, colorless
*V* = 2683.1 (2) Å^3^	0.40 × 0.30 × 0.20 mm
*Z* = 4	

### Data collection

**Table:**

Bruker SMART APEX diffractometer	6165 independent reflections
Radiation source: fine-focus sealed tube	5710 reflections with *I* > 2σ(*I*)
graphite	*R*_int_ = 0.023
ω scans	θ_max_ = 27.5°, θ_min_ = 1.8°
Absorption correction: multi-scan (*SADABS*; Sheldrick, 1996)	*h* = −12→11
*T*_min_ = 0.694, *T*_max_ = 0.827	*k* = −20→20
25326 measured reflections	*l* = −22→21

### Refinement

**Table:**

Refinement on *F*^2^	Primary atom site location: structure-invariant direct methods
Least-squares matrix: full	Secondary atom site location: difference Fourier map
*R*[*F*^2^ > 2σ(*F*^2^)] = 0.019	Hydrogen site location: inferred from neighbouring sites
*wR*(*F*^2^) = 0.049	H atoms treated by a mixture of independent and constrained refinement
*S* = 1.02	*w* = 1/[σ^2^(*F*_o_^2^) + (0.0227*P*)^2^ + 1.7091*P*] where *P* = (*F*_o_^2^ + 2*F*_c_^2^)/3
6165 reflections	(Δ/σ)_max_ = 0.001
338 parameters	Δρ_max_ = 0.35 e Å^−^^3^
1 restraint	Δρ_min_ = −0.50 e Å^−^^3^

### Fractional atomic coordinates and isotropic or equivalent isotropic displacement parameters (Å^2^)

**Table:**

	*x*	*y*	*z*	*U*_iso_*/*U*_eq_	
Sn1	0.914994 (9)	0.372319 (5)	0.750433 (5)	0.01199 (4)	
O1	0.88805 (10)	0.26134 (6)	0.67977 (6)	0.0167 (2)	
O2	0.66233 (11)	0.26088 (6)	0.67972 (6)	0.0181 (2)	
O3	0.53077 (10)	−0.00855 (6)	0.66684 (6)	0.0160 (2)	
N1	0.51088 (14)	0.12682 (7)	0.69698 (8)	0.0177 (3)	
H1	0.5391 (19)	0.1765 (7)	0.6891 (11)	0.022 (5)*	
C1	0.78221 (14)	0.45043 (8)	0.67164 (8)	0.0150 (3)	
C2	0.78027 (18)	0.44190 (10)	0.59071 (9)	0.0242 (3)	
H2	0.8315	0.3985	0.5717	0.029*	
C3	0.70416 (19)	0.49620 (11)	0.53752 (10)	0.0293 (4)	
H3	0.7034	0.4895	0.4825	0.035*	
C4	0.62957 (17)	0.55996 (10)	0.56442 (10)	0.0257 (3)	
H4	0.5788	0.5975	0.5280	0.031*	
C5	0.62943 (15)	0.56869 (9)	0.64469 (10)	0.0209 (3)	
H5	0.5781	0.6122	0.6635	0.025*	
C6	0.70430 (14)	0.51388 (9)	0.69779 (9)	0.0170 (3)	
H6	0.7024	0.5197	0.7527	0.020*	
C7	1.12405 (15)	0.37813 (8)	0.73368 (9)	0.0143 (3)	
C8	1.22988 (15)	0.39979 (9)	0.79465 (9)	0.0175 (3)	
H8	1.2096	0.4093	0.8461	0.021*	
C9	1.36463 (16)	0.40761 (10)	0.78118 (10)	0.0230 (3)	
H9	1.4356	0.4216	0.8236	0.028*	
C10	1.39583 (16)	0.39509 (9)	0.70631 (10)	0.0232 (3)	
H10	1.4880	0.4002	0.6973	0.028*	
C11	1.29187 (18)	0.37515 (9)	0.64477 (10)	0.0230 (3)	
H11	1.3124	0.3677	0.5931	0.028*	
C12	1.15697 (16)	0.36592 (9)	0.65838 (9)	0.0188 (3)	
H12	1.0866	0.3512	0.6159	0.023*	
C13	0.85936 (15)	0.32474 (8)	0.85593 (8)	0.0147 (3)	
C14	0.72102 (16)	0.32227 (9)	0.86407 (9)	0.0180 (3)	
H14	0.6523	0.3338	0.8198	0.022*	
C15	0.68262 (17)	0.30300 (10)	0.93644 (9)	0.0226 (3)	
H15	0.5880	0.3018	0.9413	0.027*	
C16	0.78136 (19)	0.28563 (10)	1.00104 (9)	0.0245 (3)	
H16	0.7549	0.2737	1.0506	0.029*	
C17	0.91974 (18)	0.28560 (10)	0.99353 (9)	0.0244 (3)	
H17	0.9878	0.2718	1.0375	0.029*	
C18	0.95815 (16)	0.30584 (9)	0.92144 (9)	0.0198 (3)	
H18	1.0528	0.3068	0.9168	0.024*	
C19	0.76680 (15)	0.22960 (8)	0.66099 (8)	0.0144 (3)	
C20	0.76215 (15)	0.15244 (9)	0.60958 (8)	0.0140 (3)	
C21	0.86142 (16)	0.15062 (10)	0.55998 (9)	0.0195 (3)	
H21	0.9268	0.1944	0.5633	0.023*	
C22	0.86751 (17)	0.08691 (10)	0.50612 (9)	0.0224 (3)	
H22	0.9354	0.0877	0.4727	0.027*	
C23	0.77383 (17)	0.02216 (9)	0.50143 (9)	0.0200 (3)	
H23	0.7757	−0.0212	0.4640	0.024*	
C24	0.67742 (15)	0.02117 (9)	0.55164 (8)	0.0161 (3)	
H24	0.6152	−0.0243	0.5490	0.019*	
C25	0.66847 (14)	0.08489 (8)	0.60619 (8)	0.0132 (3)	
C26	0.56431 (14)	0.06626 (8)	0.66012 (8)	0.0134 (3)	
C27	0.41889 (15)	0.11215 (9)	0.75476 (9)	0.0181 (3)	
H27	0.4034	0.0509	0.7585	0.022*	
C28	0.48651 (19)	0.14361 (12)	0.83514 (10)	0.0293 (4)	
H28A	0.5067	0.2037	0.8317	0.035*	
H28B	0.5746	0.1140	0.8516	0.035*	
C29	0.3924 (2)	0.12963 (12)	0.89671 (11)	0.0342 (4)	
H29A	0.3791	0.0692	0.9036	0.041*	
H29B	0.4365	0.1528	0.9481	0.041*	
C30	0.2540 (2)	0.17094 (11)	0.87143 (12)	0.0337 (4)	
H30A	0.1931	0.1578	0.9103	0.040*	
H30B	0.2664	0.2320	0.8708	0.040*	
C31	0.18684 (19)	0.14199 (13)	0.79050 (13)	0.0357 (4)	
H31A	0.1005	0.1734	0.7742	0.043*	
H31B	0.1630	0.0823	0.7930	0.043*	
C32	0.28089 (18)	0.15421 (12)	0.72883 (11)	0.0298 (4)	
H32B	0.2364	0.1305	0.6777	0.036*	
H32C	0.2952	0.2144	0.7212	0.036*	

### Atomic displacement parameters (Å^2^)

**Table:**

	*U*^11^	*U*^22^	*U*^33^	*U*^12^	*U*^13^	*U*^23^
Sn1	0.01138 (5)	0.01205 (5)	0.01288 (5)	0.00013 (3)	0.00303 (4)	0.00005 (3)
O1	0.0158 (5)	0.0156 (5)	0.0186 (5)	−0.0014 (4)	0.0025 (4)	−0.0032 (4)
O2	0.0180 (5)	0.0149 (5)	0.0229 (5)	−0.0007 (4)	0.0081 (4)	−0.0011 (4)
O3	0.0181 (5)	0.0133 (4)	0.0169 (5)	−0.0009 (4)	0.0042 (4)	0.0016 (4)
N1	0.0202 (6)	0.0132 (6)	0.0222 (6)	−0.0013 (5)	0.0109 (5)	0.0013 (5)
C1	0.0143 (7)	0.0140 (6)	0.0167 (7)	−0.0009 (5)	0.0019 (5)	0.0015 (5)
C2	0.0300 (9)	0.0236 (8)	0.0195 (8)	0.0096 (6)	0.0057 (6)	0.0005 (6)
C3	0.0373 (10)	0.0334 (9)	0.0171 (7)	0.0116 (7)	0.0040 (7)	0.0050 (7)
C4	0.0249 (8)	0.0257 (8)	0.0262 (8)	0.0068 (6)	0.0035 (6)	0.0092 (6)
C5	0.0152 (7)	0.0186 (7)	0.0296 (8)	0.0033 (5)	0.0056 (6)	0.0011 (6)
C6	0.0129 (6)	0.0201 (7)	0.0185 (7)	−0.0011 (5)	0.0038 (5)	−0.0020 (5)
C7	0.0144 (6)	0.0116 (6)	0.0175 (7)	0.0009 (5)	0.0042 (5)	0.0000 (5)
C8	0.0175 (7)	0.0176 (7)	0.0178 (7)	0.0014 (5)	0.0043 (6)	−0.0015 (5)
C9	0.0158 (7)	0.0223 (7)	0.0300 (8)	0.0001 (6)	0.0012 (6)	−0.0013 (6)
C10	0.0166 (7)	0.0176 (7)	0.0379 (9)	−0.0013 (6)	0.0124 (7)	0.0003 (6)
C11	0.0278 (8)	0.0197 (7)	0.0253 (8)	−0.0019 (6)	0.0158 (7)	−0.0014 (6)
C12	0.0203 (7)	0.0189 (7)	0.0180 (7)	−0.0022 (5)	0.0056 (6)	−0.0029 (5)
C13	0.0188 (7)	0.0112 (6)	0.0147 (6)	0.0001 (5)	0.0045 (5)	−0.0009 (5)
C14	0.0189 (7)	0.0193 (7)	0.0162 (7)	0.0014 (5)	0.0041 (5)	0.0000 (5)
C15	0.0255 (8)	0.0217 (7)	0.0235 (8)	−0.0008 (6)	0.0126 (6)	−0.0014 (6)
C16	0.0410 (10)	0.0182 (7)	0.0157 (7)	−0.0039 (6)	0.0089 (7)	0.0003 (6)
C17	0.0353 (9)	0.0184 (7)	0.0168 (7)	−0.0028 (6)	−0.0040 (6)	0.0025 (6)
C18	0.0200 (7)	0.0161 (7)	0.0222 (7)	−0.0018 (5)	0.0005 (6)	0.0007 (6)
C19	0.0185 (7)	0.0131 (6)	0.0122 (6)	−0.0005 (5)	0.0039 (5)	0.0022 (5)
C20	0.0155 (7)	0.0136 (6)	0.0130 (6)	0.0003 (5)	0.0023 (5)	0.0006 (5)
C21	0.0217 (8)	0.0189 (7)	0.0195 (7)	−0.0046 (6)	0.0078 (6)	−0.0020 (6)
C22	0.0274 (8)	0.0230 (7)	0.0200 (7)	−0.0026 (6)	0.0133 (6)	−0.0022 (6)
C23	0.0290 (8)	0.0168 (7)	0.0156 (7)	−0.0002 (6)	0.0074 (6)	−0.0027 (5)
C24	0.0194 (7)	0.0143 (6)	0.0142 (6)	−0.0014 (5)	0.0018 (5)	0.0006 (5)
C25	0.0132 (6)	0.0142 (6)	0.0120 (6)	0.0010 (5)	0.0019 (5)	0.0017 (5)
C26	0.0125 (6)	0.0151 (6)	0.0121 (6)	0.0002 (5)	0.0005 (5)	0.0020 (5)
C27	0.0206 (7)	0.0134 (6)	0.0234 (8)	0.0004 (5)	0.0126 (6)	0.0019 (5)
C28	0.0249 (9)	0.0423 (10)	0.0231 (8)	−0.0018 (7)	0.0113 (7)	−0.0007 (7)
C29	0.0366 (10)	0.0444 (11)	0.0260 (9)	−0.0012 (8)	0.0181 (8)	0.0011 (7)
C30	0.0437 (11)	0.0218 (8)	0.0447 (11)	0.0067 (7)	0.0340 (9)	0.0064 (7)
C31	0.0211 (9)	0.0427 (10)	0.0480 (12)	0.0074 (7)	0.0194 (8)	0.0164 (9)
C32	0.0207 (8)	0.0401 (9)	0.0310 (9)	0.0039 (7)	0.0118 (7)	0.0107 (8)

### Geometric parameters (Å, °)

**Table:**

Sn1—C1	2.130 (1)	C14—H14	0.9500
Sn1—C7	2.129 (2)	C15—C16	1.379 (2)
Sn1—C13	2.119 (1)	C15—H15	0.9500
Sn1—O1	2.149 (1)	C16—C17	1.391 (3)
Sn1—O3^i^	2.392 (1)	C16—H16	0.9500
O1—C19	1.2908 (17)	C17—C18	1.391 (2)
O2—C19	1.2350 (18)	C17—H17	0.9500
O3—C26	1.2574 (17)	C18—H18	0.9500
O3—Sn1^ii^	2.3917 (10)	C19—C20	1.5186 (19)
N1—C26	1.3171 (18)	C20—C21	1.399 (2)
N1—C27	1.4687 (18)	C20—C25	1.4204 (19)
N1—H1	0.864 (9)	C21—C22	1.388 (2)
C1—C6	1.395 (2)	C21—H21	0.9500
C1—C2	1.393 (2)	C22—C23	1.385 (2)
C2—C3	1.391 (2)	C22—H22	0.9500
C2—H2	0.9500	C23—C24	1.384 (2)
C3—C4	1.384 (2)	C23—H23	0.9500
C3—H3	0.9500	C24—C25	1.4004 (19)
C4—C5	1.385 (2)	C24—H24	0.9500
C4—H4	0.9500	C25—C26	1.5208 (19)
C5—C6	1.390 (2)	C27—C32	1.519 (2)
C5—H5	0.9500	C27—C28	1.517 (2)
C6—H6	0.9500	C27—H27	1.0000
C7—C8	1.395 (2)	C28—C29	1.533 (2)
C7—C12	1.397 (2)	C28—H28A	0.9900
C8—C9	1.391 (2)	C28—H28B	0.9900
C8—H8	0.9500	C29—C30	1.515 (3)
C9—C10	1.385 (2)	C29—H29A	0.9900
C9—H9	0.9500	C29—H29B	0.9900
C10—C11	1.383 (2)	C30—C31	1.511 (3)
C10—H10	0.9500	C30—H30A	0.9900
C11—C12	1.395 (2)	C30—H30B	0.9900
C11—H11	0.9500	C31—C32	1.530 (2)
C12—H12	0.9500	C31—H31A	0.9900
C13—C14	1.394 (2)	C31—H31B	0.9900
C13—C18	1.395 (2)	C32—H32B	0.9900
C14—C15	1.392 (2)	C32—H32C	0.9900
			
C13—Sn1—C7	121.55 (6)	C16—C17—H17	120.1
C13—Sn1—C1	122.49 (5)	C17—C18—C13	120.82 (15)
C7—Sn1—C1	113.32 (5)	C17—C18—H18	119.6
C13—Sn1—O1	99.06 (5)	C13—C18—H18	119.6
C7—Sn1—O1	89.59 (5)	O2—C19—O1	123.47 (13)
C1—Sn1—O1	97.16 (5)	O2—C19—C20	122.47 (13)
C13—Sn1—O3^i^	81.11 (4)	O1—C19—C20	113.98 (12)
C7—Sn1—O3^i^	85.30 (4)	C21—C20—C25	118.31 (13)
C1—Sn1—O3^i^	87.67 (4)	C21—C20—C19	114.42 (12)
O1—Sn1—O3^i^	174.06 (4)	C25—C20—C19	127.27 (12)
C19—O1—Sn1	119.52 (9)	C22—C21—C20	122.14 (14)
C26—O3—Sn1^ii^	141.59 (9)	C22—C21—H21	118.9
C26—N1—C27	123.08 (12)	C20—C21—H21	118.9
C26—N1—H1	116.4 (13)	C21—C22—C23	119.47 (14)
C27—N1—H1	120.3 (13)	C21—C22—H22	120.3
C6—C1—C2	118.35 (13)	C23—C22—H22	120.3
C6—C1—Sn1	122.69 (11)	C24—C23—C22	119.46 (13)
C2—C1—Sn1	118.80 (11)	C24—C23—H23	120.3
C3—C2—C1	120.72 (14)	C22—C23—H23	120.3
C3—C2—H2	119.6	C23—C24—C25	122.23 (13)
C1—C2—H2	119.6	C23—C24—H24	118.9
C4—C3—C2	120.30 (15)	C25—C24—H24	118.9
C4—C3—H3	119.8	C24—C25—C20	118.33 (13)
C2—C3—H3	119.8	C24—C25—C26	112.71 (12)
C3—C4—C5	119.62 (15)	C20—C25—C26	128.68 (12)
C3—C4—H4	120.2	O3—C26—N1	122.06 (13)
C5—C4—H4	120.2	O3—C26—C25	117.27 (12)
C4—C5—C6	120.08 (14)	N1—C26—C25	120.67 (12)
C4—C5—H5	120.0	N1—C27—C32	110.68 (12)
C6—C5—H5	120.0	N1—C27—C28	109.45 (13)
C1—C6—C5	120.91 (14)	C32—C27—C28	110.90 (14)
C1—C6—H6	119.5	N1—C27—H27	108.6
C5—C6—H6	119.5	C32—C27—H27	108.6
C8—C7—C12	118.07 (14)	C28—C27—H27	108.6
C8—C7—Sn1	122.05 (11)	C27—C28—C29	110.67 (15)
C12—C7—Sn1	119.72 (11)	C27—C28—H28A	109.5
C9—C8—C7	120.98 (14)	C29—C28—H28A	109.5
C9—C8—H8	119.5	C27—C28—H28B	109.5
C7—C8—H8	119.5	C29—C28—H28B	109.5
C10—C9—C8	120.32 (15)	H28A—C28—H28B	108.1
C10—C9—H9	119.8	C30—C29—C28	110.89 (15)
C8—C9—H9	119.8	C30—C29—H29A	109.5
C9—C10—C11	119.54 (14)	C28—C29—H29A	109.5
C9—C10—H10	120.2	C30—C29—H29B	109.5
C11—C10—H10	120.2	C28—C29—H29B	109.5
C10—C11—C12	120.25 (15)	H29A—C29—H29B	108.0
C10—C11—H11	119.9	C31—C30—C29	111.49 (15)
C12—C11—H11	119.9	C31—C30—H30A	109.3
C11—C12—C7	120.82 (15)	C29—C30—H30A	109.3
C11—C12—H12	119.6	C31—C30—H30B	109.3
C7—C12—H12	119.6	C29—C30—H30B	109.3
C14—C13—C18	118.56 (13)	H30A—C30—H30B	108.0
C14—C13—Sn1	119.37 (10)	C30—C31—C32	111.89 (16)
C18—C13—Sn1	121.55 (11)	C30—C31—H31A	109.2
C15—C14—C13	120.64 (14)	C32—C31—H31A	109.2
C15—C14—H14	119.7	C30—C31—H31B	109.2
C13—C14—H14	119.7	C32—C31—H31B	109.2
C16—C15—C14	120.24 (15)	H31A—C31—H31B	107.9
C16—C15—H15	119.9	C27—C32—C31	110.81 (14)
C14—C15—H15	119.9	C27—C32—H32B	109.5
C15—C16—C17	119.93 (14)	C31—C32—H32B	109.5
C15—C16—H16	120.0	C27—C32—H32C	109.5
C17—C16—H16	120.0	C31—C32—H32C	109.5
C18—C17—C16	119.78 (15)	H32B—C32—H32C	108.1
C18—C17—H17	120.1		
			
C13—Sn1—O1—C19	64.82 (11)	C18—C13—C14—C15	1.3 (2)
C7—Sn1—O1—C19	−173.26 (10)	Sn1—C13—C14—C15	−170.53 (11)
C1—Sn1—O1—C19	−59.80 (10)	C13—C14—C15—C16	−0.4 (2)
C13—Sn1—C1—C6	41.13 (14)	C14—C15—C16—C17	−1.4 (2)
C7—Sn1—C1—C6	−120.66 (12)	C15—C16—C17—C18	2.1 (2)
O1—Sn1—C1—C6	146.69 (11)	C16—C17—C18—C13	−1.2 (2)
O3^i^—Sn1—C1—C6	−36.79 (12)	C14—C13—C18—C17	−0.5 (2)
C13—Sn1—C1—C2	−143.59 (12)	Sn1—C13—C18—C17	171.13 (11)
C7—Sn1—C1—C2	54.62 (13)	Sn1—O1—C19—O2	2.02 (18)
O1—Sn1—C1—C2	−38.03 (13)	Sn1—O1—C19—C20	178.78 (8)
O3^i^—Sn1—C1—C2	138.49 (12)	O2—C19—C20—C21	146.62 (14)
C6—C1—C2—C3	1.0 (2)	O1—C19—C20—C21	−30.18 (18)
Sn1—C1—C2—C3	−174.49 (14)	O2—C19—C20—C25	−32.3 (2)
C1—C2—C3—C4	0.3 (3)	O1—C19—C20—C25	150.90 (14)
C2—C3—C4—C5	−1.0 (3)	C25—C20—C21—C22	2.4 (2)
C3—C4—C5—C6	0.3 (3)	C19—C20—C21—C22	−176.59 (14)
C2—C1—C6—C5	−1.7 (2)	C20—C21—C22—C23	−0.8 (2)
Sn1—C1—C6—C5	173.61 (11)	C21—C22—C23—C24	−1.3 (2)
C4—C5—C6—C1	1.1 (2)	C22—C23—C24—C25	1.7 (2)
C13—Sn1—C7—C8	−38.88 (13)	C23—C24—C25—C20	−0.1 (2)
C1—Sn1—C7—C8	123.10 (11)	C23—C24—C25—C26	−174.48 (13)
O1—Sn1—C7—C8	−139.28 (11)	C21—C20—C25—C24	−2.0 (2)
O3^i^—Sn1—C7—C8	37.67 (11)	C19—C20—C25—C24	176.91 (13)
C13—Sn1—C7—C12	145.85 (10)	C21—C20—C25—C26	171.43 (14)
C1—Sn1—C7—C12	−52.17 (12)	C19—C20—C25—C26	−9.7 (2)
O1—Sn1—C7—C12	45.45 (11)	Sn1^ii^—O3—C26—N1	−85.49 (19)
O3^i^—Sn1—C7—C12	−137.60 (11)	Sn1^ii^—O3—C26—C25	95.48 (16)
C12—C7—C8—C9	−1.1 (2)	C27—N1—C26—O3	6.3 (2)
Sn1—C7—C8—C9	−176.43 (11)	C27—N1—C26—C25	−174.74 (13)
C7—C8—C9—C10	0.9 (2)	C24—C25—C26—O3	20.40 (18)
C8—C9—C10—C11	0.3 (2)	C20—C25—C26—O3	−153.31 (14)
C9—C10—C11—C12	−1.4 (2)	C24—C25—C26—N1	−158.65 (13)
C10—C11—C12—C7	1.2 (2)	C20—C25—C26—N1	27.6 (2)
C8—C7—C12—C11	0.0 (2)	C26—N1—C27—C32	−121.11 (16)
Sn1—C7—C12—C11	175.48 (11)	C26—N1—C27—C28	116.37 (16)
C7—Sn1—C13—C14	179.98 (10)	N1—C27—C28—C29	179.65 (14)
C1—Sn1—C13—C14	19.66 (13)	C32—C27—C28—C29	57.26 (19)
O1—Sn1—C13—C14	−84.89 (11)	C27—C28—C29—C30	−56.6 (2)
O3^i^—Sn1—C13—C14	101.13 (11)	C28—C29—C30—C31	55.2 (2)
C7—Sn1—C13—C18	8.41 (14)	C29—C30—C31—C32	−54.5 (2)
C1—Sn1—C13—C18	−151.91 (11)	N1—C27—C32—C31	−177.73 (15)
O1—Sn1—C13—C18	103.54 (11)	C28—C27—C32—C31	−56.1 (2)
O3^i^—Sn1—C13—C18	−70.44 (11)	C30—C31—C32—C27	54.7 (2)

Symmetry codes: (i) −*x*+3/2, *y*+1/2, −*z*+3/2; (ii) −*x*+3/2, *y*−1/2, −*z*+3/2.

### Hydrogen-bond geometry (Å, °)

**Table:**

*D*—H···*A*	*D*—H	H···*A*	*D*···*A*	*D*—H···*A*
N1—H1···O2	0.86 (1)	1.85 (1)	2.666 (2)	158 (2)
